# Novel case of laparoscopically resected gastric adenocarcinoma concurrent with lanthanum deposition

**DOI:** 10.1002/ccr3.6497

**Published:** 2022-12-27

**Authors:** Kosuke Hirose, Shunichi Saito, Yumi Oshiro, Kazuhito Minami, Koji Ikegami, Koichi Kurahara, Takuya Honbo, Noriaki Sadanaga, Hiroshi Matsuura

**Affiliations:** ^1^ Department of Surgery Saiseikai Fukuoka General Hospital Fukuoka Japan; ^2^ Department of Surgery Matsuyama Red Cross Hospital Matsuyama Japan; ^3^ Department of Surgery and Science, Graduate School of Medical Sciences Kyushu University Fukuoka Japan; ^4^ Department of Anatomic Pathology and Pathological Sciences Matsuyama Red Cross Hospital Matsuyama Japan; ^5^ Department of gastroenterology Matsuyama Red Cross Hospital Matsuyama Japan

**Keywords:** concurrent carcinoma, gastric cancer, lanthanum deposition, laparoscopic surgery

## Abstract

A 73‐year‐old man taking lanthanum carbonate for hemodialysis showed progressing gastric mucosal changes with lanthanum deposition. Regular examination revealed concurrent gastric carcinoma. The extent and depth of its invasion were ambiguous because of the surrounding lanthanum deposition. Furthermore, there could be other potent carcinomas, and curative laparoscopic gastrectomy was performed.

## Graphical abstract

It is difficult to determine the clinical depth and extent of involvement by the gastric carcinoma in cases of lanthanum deposits with hemodialysis. Such cases require more careful preoperative evaluation and therapeutic strategy.

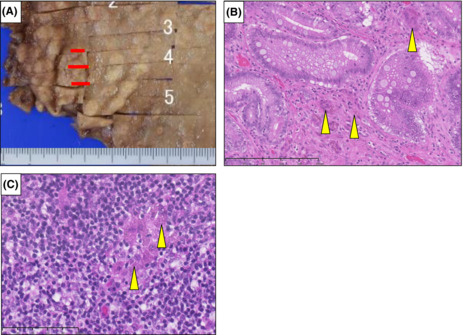

## 1 INTRODUCTION

Lanthanum carbonate is one of the widely used phosphate binders that is taken orally to reduce the serum phosphate levels in patients with chronic kidney disease undergoing dialysis.^1^ Lanthanum carbonate binds to dietary phosphate to form insoluble complexes that are excreted via the fecal route; however, recently lanthanum carbonate deposition has been reported in the gastric and duodenal mucosa of patients taking these medications.^2^, ^3^, ^4^, ^5^, ^6^, ^7^, ^8^

The endoscopic findings of gastric lanthanum deposition have been described as white lesions, elevations, erosions, and ulcerations. Moreover, sometimes those features might resemble those of carcinoma. In such cases of gastric cancer with lanthanum deposition, careful pretherapeutic diagnosis is warranted.

Pathologically, lanthanum depositions usually appear as subepithelial collections of histiocytes or small foreign body granulomas, accompanied by intestinal metaplasia, regenerative changes, and foveolar hyperplasia.^1^, ^9^, ^10^, ^11^ Although some studies have reported that lanthanum deposition and subsequent mucosal changes potentially induce neoplastic changes in lesions, the relationship between lanthanum deposition and carcinogenesis is unknown.^11^, ^12^

There are also few reports regarding gastric cancer concurrent with lanthanum deposition^2^, ^12^, ^13^, ^14^ in which surgical resection was performed.^2^, ^12^

Unlike previous cases in which surgical resection was performed, in this case, it was difficult to clearly determine the depth and extent of invasion by gastric adenocarcinoma; however, the surrounding ambiguous mucosal changes with lanthanum deposition were clearly observed. Further, such cases require careful diagnosis and a definitive therapeutic strategy. Herein, we present a case of laparoscopically resected gastric adenocarcinoma with concurrent lanthanum deposition in which the depth and extent of invasion by the tumor was difficult to determine for precise preoperative diagnosis.

## 2 CASE HISTORY

A 73‐year‐old man had been on peritoneal dialysis for 9 years for chronic renal failure due to immunoglobulin A nephropathy and had switched to hemodialysis 3 years before the surgeries.

**FIGURE 1:**
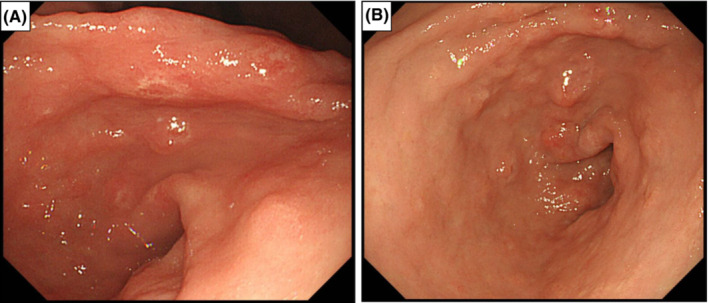
(A) Endoscopic image of lanthanum deposition in the stomach few months after drug initiation. (B) Follow‐up endoscopic image of lanthanum deposition at the same site of the lesions.

**FIGURE 2:**
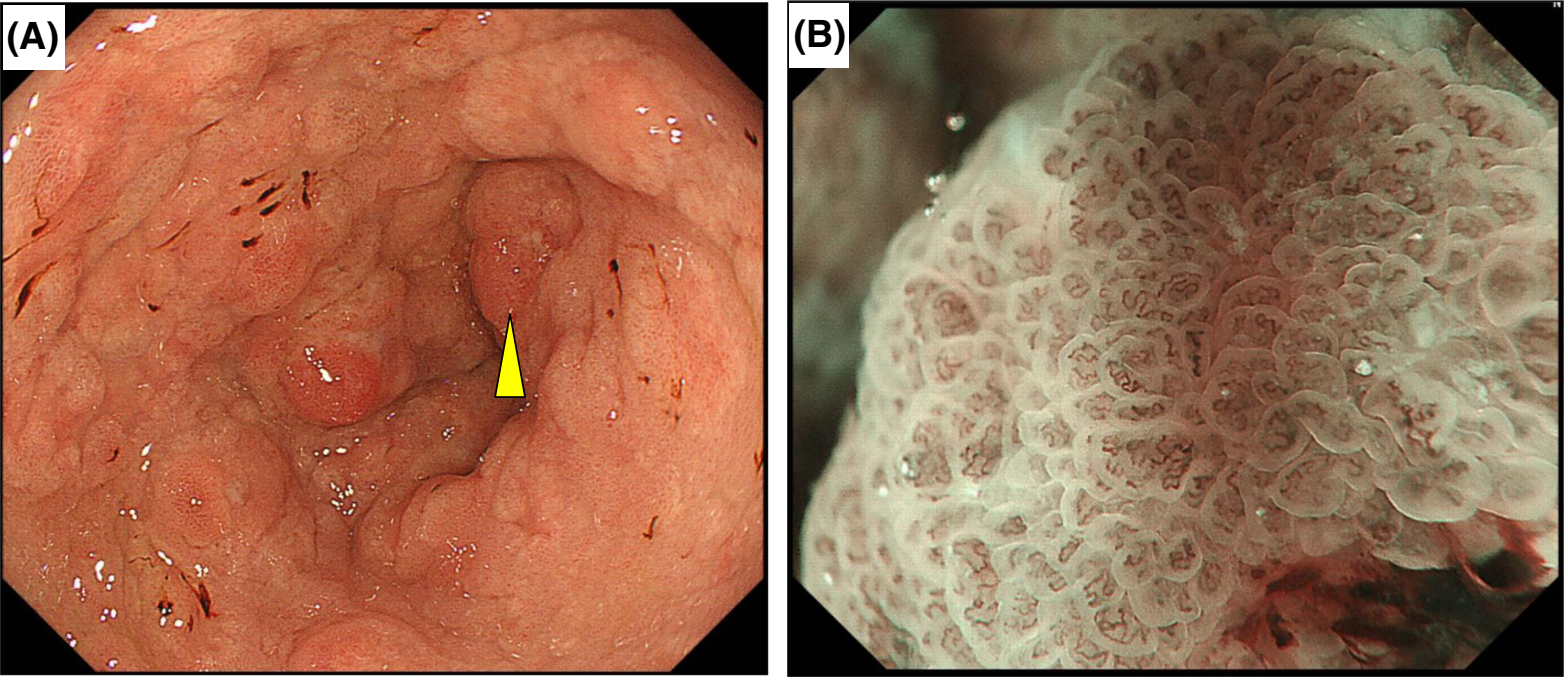
(A) Endoscopic images of grown lanthanum deposition and gastric cancer at antrum (arrowhead). (B) Narrow band imaging of cancer showing no irregular microvascular pattern.

**FIGURE 3:**
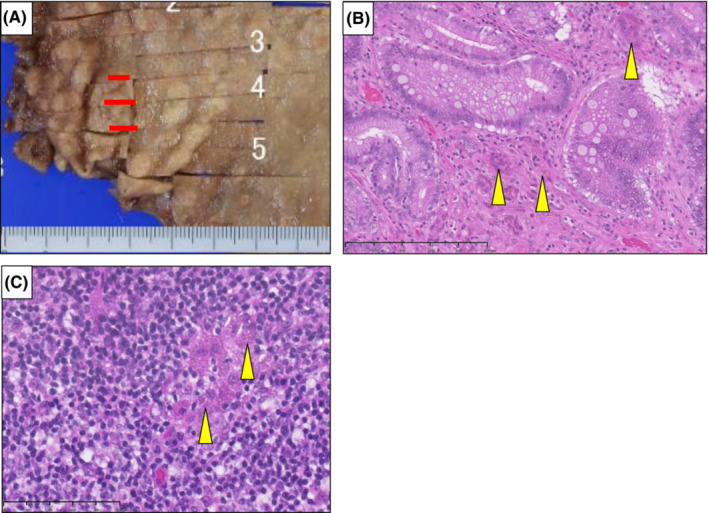
(A) Resected specimen and mapping of the malignant region (yellow line). (B) Histological image of gastric adenocarcinoma with hematoxylin and eosin staining (arrowheads indicate lanthanum carbonate eaten by histiocytes). (C) Histological image of a resected lymph node. It also includes histiocytes eating lanthanum carbonate (arrowhead).

**FIGURE 4:**
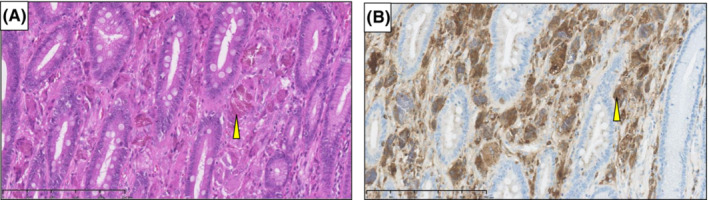
Histological images indicate the distribution of CD68‐positive macrophages eating lanthanum carbonate with hematoxylin and eosin staining (A). Immunohistochemical staining with CD68 monoclonal antibody for the same specimen (B)

He had been taking lanthanum carbonate for 7 years. Several months after lanthanum carbonate initiation, regular endoscopy revealed white granular lesions on the mucosa of the gastric body and fundus (Figure 1A). The results of a biopsy of these lesions revealed chronic active inflammation with regenerative epithelium, intestinal metaplasia, and lanthanum deposition. Regular annual endoscopy revealed that these lesions had grown, changed morphologically, and spread gradually (Figure 1B), but they showed no malignancy. Two months before the surgery, regular endoscopy revealed a gradually growing, reddish, and elevated mucosa that was surrounded by white granular mucosa in the gastric antrum (Figure 2A). No apparent superficial mucosal changes, including ulcers, were observed and narrow band imaging (NBI) showed no signs of malignancy, such as an irregular microvascular pattern (Figure 2B). Although endoscopic ultasonography was not performed, the gastroenterologists performed the biopsy cautiously, and the histological examination revealed well‐differentiated adenocarcinoma with a diameter of ≤2 cm. Considering the clinical findings, the clinical stage was classified as T1bN0M0 stage I based on the TNM classification specified by the International Union Against Cancer (UICC). In addition to the clinical depth of the tumor classified as T1b, the boundary between the adenocarcinoma and mucosal changes with lanthanum deposition was unclear by endoscopy. Moreover, potential neoplastic lesions with lanthanum deposition might occur in the gastric body. Therefore, it was considered irrelevant to choose endoscopic resection, and we planned to perform curative resection with adequate margins. Although the patient was undergoing hemodialysis and a high‐surgical risk was involved; performance status was 0; and the organ function was preserved except for the renal function, the risk of general anesthesia and surgery were not considered critical. Considering the high‐surgical risk associated with hemodialysis carefully, we chose laparoscopic distal gastrectomy with lymph node dissection. Postoperative histopathological examination revealed a well‐differentiated tubular adenocarcinoma, which was limited to the antrum and surrounded by lanthanum nodules (Figure 3A). Its pathological diameter was 15 × 6 mm. Ulcer and vessel invasion were not observed. The tumor was limited to the mucosa at the gastric antrum, and the pathological stage was classified as T1aN0M0 stage I. Lanthanum deposition was observed in the lamina propria of the carcinoma and gastric mucosa (Figure 3B). Histological alterations, such as glandular atrophy and hyperplasia, were also observed. The resected lymph nodes also included lanthanum deposits eaten by macrophages (Figure 3C). In the interstitium of cancerous and non‐cancerous lesions, lanthanum was mostly eaten by macrophages that expressed CD68 (Figure 4A,B).

## 3 DISCUSSION

We report a rare case of laparoscopically resected gastric adenocarcinoma with concurrent lanthanum deposition. In this case, we chose surgical resection because of the clinical depth of the tumor which was classified as T1b; possibility of occurrence of other potential carcinomas with lanthanum deposition; and ambiguous boundary between the malignant tissue and mucosal changes with lanthanum deposition.

There are a few reports of surgically resected gastric cancer with lanthanum deposition, as summarized in Table 1.^2^, ^12^ Yabuki et al.^11^ reported a summary of three patients with gastric cancer with concurrent lanthanum deposition who underwent surgical resection. They reported that the duration of lanthanum administration ranged from 3 to 36 months, and the depth of tumor was the entire submucosa (range: 1.5–5 mm). Lanthanum deposition was identified in the regional lymph nodes to some degree in all the three cases. These findings support the hypothesis that lanthanum malabsorption in the gastric mucosa leads to other lymph nodes via lymphatic flow. Tonooka et al.^12^ reported a case of multiple but early gastric cancers in a patient with lanthanum deposition who underwent subtotal gastrectomy after 7 months of drug use. In this case, many macrophages with lanthanum deposits accumulated in the lamina propria of the gastric wall and tumors, which was confirmed by scanning electron microscopy‐energy‐dispersive X‐ray spectroscopy (SEM‐ESD). They also showed the intestinal metaplasia associated with lanthanum deposition and considered that altered tight junction proteins may lead to its permeability, possibly resulting in lanthanum deposition. Our case showed mucosal atrophy and metaplasia, including a cancerous lesion at the gastric antrum, with CD68‐positive macrophages containing lanthanum deposits, which were also identified in the regional lymph nodes.

**TABLE 1:** Summary of cases reporting surgically resected gastric carcinoma with lanthanum deposition, including this case.

Case	Age/sex	Location	Histology	Differentiation	pT	pN	Dose of LC (mg/day)	Duration of LC administration (months)	*Helicobacter pylori* infection	PPI	Laparoscopic surgery	Reseced range	Prognosis	Author
1	81/F	Multiple	Adeno.	Well	T1a	0	750	7	NA	NA	No	Subtotal	3 years alive No reccurence	Tonooka et al
2	77/F	Antrum	Adeno.	Well	T1b	0	1500	36	Negative	Use	Yes	Distal	Unknown	Yabuki et al
3	68/F	Body	Adeno.	Por/sig	T1b	0	750	3	NA	Use	Yes	Distal	Unknown	Yabuki et al
4	77/M	Antrum	Adeno.	Well	T1b	1	750	12	NA	Not use	Yes	Distal	Unknown	Yabuki et al
5	73/M	Antrum	Adeno.	Well	T1a	0	750	84	Negative	Use	Yes	Distal	8 months alive No reccurence	our case

Abbreviations: Adeno, adenocarcinoma; LC, lanthanum carbonate; NA, not available; PPI, proton pump inhibitor.

In gastric adenocarcinoma concurrent with lanthanum deposition, the findings of lanthanum deposition by endoscopy are occasionally similar to carcinoma, and it might be difficult to diagnose the precise depth and extent of the tumor and mark the boundary between the neoplasm and lesions. The findings of lanthanum deposition have been usually described as shiny, bright white, and of varied sizes and shapes, from small and flat to elevated plaques.^9^ Gastric carcinoma shows variable findings with endoscopy. In previous reports, early gastric cancer with lanthanum deposition was observed as a depressed area surrounded by annular whitish lanthanum‐deposited mucosa^8^, ^13^ and resected by endoscopy. Yabuki et al.^11^ summarized the cases of three patients with concurrent gastric cancer who underwent laparoscopic distal gastrectomy, but the endoscopic findings showed a clear boundary between the malignant tissue and surrounding mucosa with lanthanum deposition. Tonooka et al.^12^ also reported concurrent gastric cancer with lanthanum deposition in patients receiving subtotal gastrectomy, but they did not refer to the endoscopic findings. The usefulness of magnified imaging using NBI was recently reported, and it shows irregular vascular and pit structures of malignant tissues.^13^ However, in this case, the carcinoma was detected within multiple reddish granular areas with lanthanum deposition without any specific malignant findings observed with NBI, and it was difficult to determine the depth and extent of invasion by the tumor and define the boundary between the neoplasm and lanthanum deposition. In addition, the occurrence of other potential carcinomas with lanthanum deposition cannot be ruled out. Therefore, we were concerned about the possibility of noncurative resection with endoscopy and other lurking regions, and the carcinoma was surgically resected. Furthermore, using laparoscopy made it possible to observe the entire serous membrane of the stomach, nearby lymph nodes, and other organs in detail.

The relationship between malignancy and lanthanum deposition in gastric cancer concurrent with lanthanum deposition is unknown. Yabuki et al.^11^ examined the effect of oral administration of lanthanum carbonate on gastric mucosa in a rat model. They showed that it caused various histologic alterations, such as glandular atrophy, the proliferation of mucous neck cells, and intestinal metaplasia, and concluded that these mucosal injuries, named lanthanum gastropathy, could potentially induce abnormal cell proliferation or neoplastic lesions. In sequential changes of the gastric epithelium due to lanthanum gastropathy, it becomes difficult to identify the boundary between the neoplasm and mucosal changes without malignancy, and surgical resection is recommended in such cases.

Herein, we presented a rare case of laparoscopically resected gastric adenocarcinoma concurrent with lanthanum deposition because of the difficulties in diagnosing the precise clinical depth and extent of the tumor, in determining the possibility of occurrence of other neoplastic lesions with lanthanum deposition, and in defining the ambiguous boundary between the carcinomatous and noncarcinomatous changes in the lesion. The clinical significance and relationship between neoplasm and lanthanum deposition have been suggested, but enough evidence has not been reported. Our results add to this growing body of data and will aid in clarifying this relationship.

## AUTHOR CONTRIBUTIONS

KH performed the surgery and observed the patients, selected the associated data, and edited the manuscript accordingly. SS also performed the surgery and took care of the patient. YO analyzed the specimens and supervised the pathology. KM performed the surgery on the patient as a supervisor. KI and KK performed the treatment of the patient as gastroenterologists. TH, NS, TN, and HM supervised and revised the article. All the authors have read and approved the final manuscript.

## FUNDING INFORMATION

This research received no specific grants from any funding agency in the public, commercial, or not‐for‐profit sectors.

## CONFLICT OF INTEREST

All the authors declare no competing interests.

## ETHICAL APPROVAL

This study was performed in accordance with the ethical standards of the Japan Surgical Society.

## CONSENT

Written informed consent was obtained from the patient for publication of this case report and accompanying dates and images.

## Data Availability

Data sharing is not applicable to this article, as no datasets were generated or analyzed during the current study.
